# Actinic Keratosis and Non-Invasive Diagnostic Techniques: An Update

**DOI:** 10.3390/biomedicines6010008

**Published:** 2018-01-08

**Authors:** Alice Casari, Johanna Chester, Giovanni Pellacani

**Affiliations:** Clinica Dermatologica, Policlinico di Modena, Via del Pozzo 70, 41124 Modena, Italy; johanna.chester@gmail.com (J.C.); pellacani.giovanni@gmail.com (G.P.)

**Keywords:** actinic keratosis, dermoscopy, confocal laser microscopy, optical coherence tomography

## Abstract

Actinic keratosis represents the earliest manifestation of non-melanoma skin cancer. Because of their risk of progression to invasive squamous cell carcinoma, an earlier diagnosis and treatment are mandatory. Their diagnosis sometimes could represent a challenge even for expert dermatologists. Dermoscopy, confocal laser microscopy and optical coherence tomography could help clinicians in diagnosis.

## 1. Introduction

Actinic keratosis (AK), the most common in situ cancerous skin lesion, was first described by Dubreuillh over one hundred years ago [1]. AKs are caused by chronic exposure to ultraviolet rays from sunlight [2,3]. The lesions have not yet acquired the full complement of chromosomal aberrations and invasive growth characteristics that are associated with invasive squamous cell carcinoma (SCC), and are considered the most important predisposing factor for SCC [4].

The prevalence of AK is highest among regions with high UV exposure. Australia, the country with the highest skin cancer rate worldwide, has an AK prevalence rate among middle age adults (>40 years old) ranging from 40% to 60%. AK prevalence is also influenced by gender; according to a study conducted in Queensland, Australia, 55% of men compared with only 37% of women aged between 30 and 70 years have been diagnosed with AK [5]. These sex-related differences have been largely attributed to a higher occupational sun exposure among men [5,6] but very recent data has suggested an alternative hypothesis [7]. Nonsteroidal anti-inflammatory drug monthly intake among fertile women over decades could play a role in AK prevention in women [8].

Three different pathways have been described for AKs: regression, persistence, or progression toward in situ or invasive SCC. Although the actual risk for an individual AK progressing to invasive SCC is unclear, estimations vary from as low as 0.1% to as high as 20% [9,10].

Even with a low individual rate of progression, patients with multiple AKs (i.e., more than 10) may have a 14% cumulative probability of developing an SCC, either within the AK or de novo, within 5 years [9]. The relative risk of SCC also increases with the number of AKs; around 1% for patients with 5 or fewer AK lesions and up to 20% for patients with more than 20 AK lesions [11]. Further evidence of the link between AK and SCC is provided by data demonstrating that up to 82% of SCCs arise within, in close proximity to, or contiguous with an AK [12,13]. However, not all SCCs arise from AK lesions, and around 40% of SCC develops on previously normal skin [14]. Whether it is cost-effective to treat all AK lesions for SCC prevention is unclear [14].

Multiple lesions, both clinically observable and subclinical, may exist across the entire areas of sun-damaged skin as a result of UV-induced field cancerization. AK share many similar molecular and histological features with SCC, and it can sometimes be difficult to clinically distinguish between the two. Clinical presentation of AK is often widely variable, and although several symptoms (e.g., bleeding, tenderness, and size) suggest more invasive disease, certain diagnosis requires histopathological examination of a biopsy sample. Multiple AKs would therefore require multiple biopsies which are not always feasible for timing, costs and aesthetic restriction due to scars that a biopsy leaves. The developments of noninvasive optical techniques, such as confocal laser microscopy (RCM) and optical coherence tomography (OCT), may reduce the requirement for invasive diagnoses.

## 2. Clinical Aspect of Actinic keratosis (AK)

AKs frequently present as multiple, ill-defined palpable macules, papules or plaques, and vary in size from a few millimeters to 1–2 cm. AKs are pinkish to red-brown, with dry, adherent scales [4]. AKs are most commonly located on the face, ears, neck, bald scalp, extensor surface of the extremities and lower lip. AKs are usually asymptomatic although some patients report itching, burning or a splinter-like sensation in the affected skin area.

In 2007, a clinical classification for grading AK (grades 1, 2, and 3) was developed [15]; grade 1 describes slightly palpable AKs (better felt than seen), grade 2 are moderately thick AKs (easily felt and seen), and grade 3 are very thick, hyperkeratotic, and/or obvious AKs. A similar scheme for the dermoscopic, confocal, and histologic grading of AKs has also been established. The clinical diagnosis between grade 3 AK and early invasive SCC, however, is subject to variable clinical interpretation. 

Clinical aspects alone are insufficient for correct AK diagnosis. Small lesion details useful for correct diagnosis and selection of treatment cannot be seen by the naked eye alone. Dermoscopy is the first instrument that is nowadays used by all dermatologists in daily practice.

## 3. Dermoscopic Aspects of AKs

Dermoscopy permits the visualization of skin structures with polarized light at a 6- to 100-fold magnification, reaching the depth of the papillary dermis [16]. The usefulness of dermoscopy in differentiating melanocytic from non-melanocytic pigmented skin lesions, both benign and malignant, is widely recognized [17,18].

The three different clinical grades of AK correspond dermoscopically to three different patterns [18,19]: Grade 1 AKs are typified by red pseudo-network pattern and discrete white scales; grade 2 corresponds to an erythematous background intermingled by white to yellow, keratotic, and enlarged follicular openings (reminiscent of the surface of a strawberry this pattern has been termed “strawberry pattern”); and grade 3 AKs exhibit either enlarged follicular openings filled with keratotic plugs over a scaly and white-yellow-appearing background or marked hyperkeratosis seen as white-yellow structureless areas. The diagnostic sensitivity and specificity of dermoscopy in AK diagnosis has been reported to reach 98% and 95%, respectively [19].

**Figure 1:**
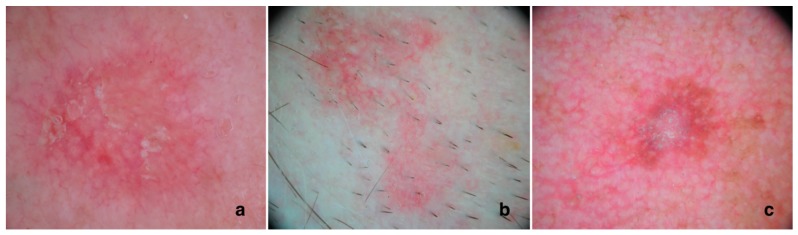
Dermoscopy of AKs. (**a**) Actinic keratosis showing a typical strawberry pattern with scales: erythema, revealing a marked pink-to-red “pseudo-network” surrounding the hair follicles; white-to-yellow surface scale; hair follicle openings filled with yellowish keratotic plugs and/or surrounded by a white halo; (**b**) Actinic keratosis showing a typical strawberry pattern without scales: erythema, revealing a marked pink-to-red “pseudo-network” surrounding the hair follicles and hair follicle openings filled with yellowish keratotic plugs and/or surrounded by a white halo; (**c**) Lightly pigmented actinic keratosis with a superficial brown network consisting of brown, curved double lines that surround enlarged, partially confluent, keratotic follicles of various sizes. This pattern is associated with a red pseudo-network and scales.

Correct non-pigmented, facial actinic keratosis clinical and dermoscopic diagnosis represents a challenge even for expert dermatologists. In facial AKs four dermoscopic features have been described: erythema, a strong pink-to-red “pseudo-network” surrounding the hair follicles; surface scale white-to-yellow; fine, linear-wavy vessels surrounding the hair follicles; and yellowish keratotic plugs in the hair follicle openings and/or surrounded by a whitish halo [19]. In 95% of cases, these features combined produce a type of “strawberry” appearance (Figure 1).

At times, AK may also show pigmentation. Zalaudek et al. [20] showed that the most typical dermoscopic picture of pigmented AK (pAK) is typified by a superficial brown network consisting of brown, curved double lines that surround enlarged, partially confluent, keratotic follicles of various sizes. A pseudo-network can be detected in benign or malignant lesions on facial skin; only the characteristics of the meshes and holes may make a definite diagnosis possible. At times, this pattern can be associated with a red pseudo-network and scales. In addition to these typical patterns, pAK, as well as lichenoid AK, may sometimes reveal pigmented structures that overlap with those of lentigo maligna [21].

These structures include grey dots (annular granular pattern), grey-brown lines (pseudo-network or rhomboidal structures) around the follicular openings and asymmetrical, pigmented follicular openings. As these features are associated with lentigo maligna (LM), a biopsy is required for differential diagnosis. Due to the overlapping patterns with LM, the diagnostic sensitivity and specificity for pAK is much lower than for non-pAK.

Kelati et al. [22] designed a study including 232 cases of pigmented lesions of the face, of which around half were malignant. They wanted to describe different dermoscopic patterns of pAK correlated with clinical patient characteristics and the evolutionary stage of the lesion. Dermoscopic patterns were divided into two categories: the follicle surroundings’ abnormalities (FSA) and follicular keratosis’ abnormalities (FKA). Besides these criteria, two newly described dermoscopic signs were used in their study, namely, the prominent central hyperkeratosis and the double white clod. FSA and FKA dermoscopic patterns were related to male gender. Central crusts and scales were related to thick plaques and the star-like appearance to hypertrophic pAK. The presence of 2 or more dermoscopic signs in both FSA and FKA was noticed in 99.1% of lesions.

Later, Lallas et al. [23] designed a study to investigate the diagnostic accuracy of established dermoscopic criteria for LM and pAK. The study sample consisted of 70 LMs, 56 pAKs and 18 solar lentigo/seb keratosis (SL/SKs). They showed that white and evident follicles, scales and red color represent significant diagnostic clues for pAK. Conversely, intense pigmentation and grey rhomboidal lines appear highly suggestive of LM.

Based on the morphological differences between the different stages of keratinocyte skin cancer, a progression model of AK developing toward SCC has been proposed, whereby the development of initially dotted or glomerular vessels, and later hairpin and linear vessels, indicate progression toward a more aggressive, invasive growth of SCC [19].

Zalaudek et al. sought to determine the dermoscopic features of facial AK, intraepidermal carcinoma (IEC), moderately to poorly differentiated invasive SCC, and well-differentiated SCC of the keratoacanthoma type [19]. A total of 243 lesions were analyzed. In line with their previous study [19] the authors found the red pseudo-network significantly associated with AK. In contrast, rosettes, a recently described AK feature, only visible with polarized light dermoscopy [24,25,26] were very rare in the study sample, despite the frequent use of polarized light dermoscopy devices. Dotted/glomerular vessels, diffuse yellow opaque scales, and microerosions were significantly more prevalent among IEC. This data suggest that diffuse peripheral erythema and a red starburst pattern may be of further aid in distinguishing AK from IEC. However, both criteria seem insufficient to differentiate between in situ and invasive SCC, as they appeared in similar frequency among both types of lesions. 

Dermoscopic features associated with invasive SCC are hairpin and/or linear-irregular vessels, targetoid hair follicles, white structureless areas, a central mass of keratin, and ulceration. These similar patterns can also be observed among keratoacanthomas. The authors also proposed a progression model of facial AKs developing into IEC and invasive SCC, similar to that previously proposed for LM [23,27].

Changes in neovascularization represents the main feature of AKs that develop into SCC in situ, and can be used for differential diagnosis. The increasing atypia is usually associated with dotted vessels around follicles in severe atypia AKs; when the dotted vessels appear to enlarge, become more convoluted (i.e., forming glomerular vessels) and clustered, and the follicles in this area miniaturize and disappear the AKs develop into an in situ SCC. With the progression to invasive SCC, the lesion thickens clinically while dermoscopically hairpin and/or linear-irregular vessels will appear: sometimes a central mass of keratin forms and ulceration may occur. The authors hypothesize that a red starburst pattern on dermoscopy might provide a dermoscopic clue for more aggressive lesions. Interestingly, the authors reported that a starburst pattern is usually not associated with stable lesions.

Occasionally a differential diagnosis is not able to be made by dermoscopy alone. In this case, a biopsy of a segment of a lesion is often required for hystological confirmation before non-surgical treatment can be performed. Other noninvasive diagnostic techniques, such as confocal laser microscopy and optical coherence tomography, are less frequently available, but where present can be very helpful in the study of the entire lesion, and can in some cases assist in differential diagnosis of AK.

## 4. Fluorescence Techniques Detection in Skin Cancer

The topical application of aminolevulanic acid (ALA) associated with photodynamic therapy, a noninvasive therapy, is widely used to treat both cancerous, that is basal cell carcinoma (BCC), and in situ squamosus cell carcinoma (SCC), and precancerous lesions, that is AK. Photodynamic therapy involves the activation of a photosensitizing drug by visible light to produce activated oxygen species within target cells, resulting in their destruction. Commonly used topical photosensitizers are aminolevulinic acid (ALA) and the methyl ester of ALA (MAL), which act as precursors of the endogenous photosensitizer protoporphyrin IX (PpIX). The target aspect is that ALA is preferentially converted into prooporphyrin IX (PpIX) in neoplastic cells.

In addition to its therapeutic uses, fluorescence emitted by MAL-induced PpIX may be useful in providing a fluorescence diagnosis of cutaneous lesions, both BCC, SCC in situ and AKs. Tumors can be delineated with the use of a Woods ultraviolet lamp, and altered skin exhibits pink fluorescence due to the presence of activated PpIX by Woods Ultraviolet light. Fluorescence can permit the detection of otherwise occult areas of abnormal skin [28].

To monitor the amount of PpIX in tissues, techniques have been developed to measure PpIX-specific fluorescence, which is important in monitoring the abundance and location of the PpIX. Noninvasive devices, such as (i) fiber optic probe/point spectrofluorometry and (ii) wide-field camera-based imaging were developed to monitor PpIX in superficial tissues. While the acquisition of information at greater tissue depths can be made with multimodal techniques, such as ultrasound (US), optical coherence tomography (OCT) and multiphoton tomography (MPT). However, fluorescence detection is not specific for AK, and although it can detect BCC, a clear distinction between the two pathologies is not possible [29].

## 5. Confocal Aspects of AKs

RCM is an in vivo, noninvasive technique with a resolution close to histology. RCM works on reflectance, scattering, and absorption of near-infrared light [16]. Hyperkeratotic lesions cannot be explore by RCM because of the shallow RCM penetration [30]. The main RCM features of AKs are superficial disruption, architectural disarray, and cellular pleomorphism at the spinous and granular layers, which yield sensitivity and specificity values of 80% and 98.6% respectively. RCM reveals a horizontal section, so a vertical invasion of the lesion cannot be visualized [30].

AK is characterized by the presence of surface scales, parakeratosis and an irregular honeycombed pattern of the spinous-granular layers. At the DEJ, small regular dermal papillae are usually observed [31,32].

**Figure 2:**
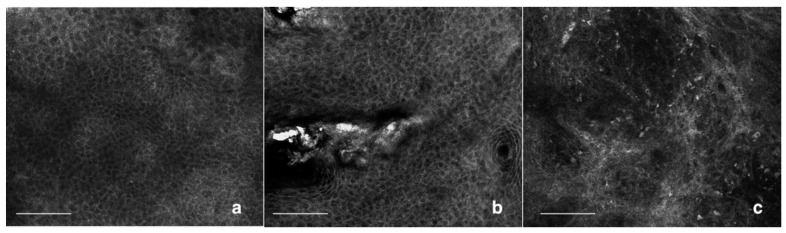
Confocal laser microscopy of AKs, epidermal layer. (**a**) Grade 1 AK presents focal areas of atypical honeycombed pattern at the level of the stratum spinosum, intermingled with areas of preserved, typical honeycombed pattern; (**b**) Grade 2 AK presents a more diffuse keratinocytes’ atypia. Keratinocytes present a marked atypia, with different cell sizes and shapes; (**c**) Grade 3 AK is characterized by a markedly atypical honeycombed pattern with areas of partial disruption of the normal epidermal layers, defined as a disarranged pattern. Pleomorphic keratinocytes show a wide variability in cellular size and shapes, and irregular intercellular keratinocyte connections are detected. The small brighter particle are lymphocytes which suggests a marked inflammatory infiltrate within the lesion. Scale bar = 50 um.

A recent study by Pelican et al. showed a significant correlation between the grading of keratinocyte atypia in AK, by experienced blinded RCM observers, and histopathological examination [33]. Based on this, Zalaudek et al. [19] showed preliminary data to correlate RCM and dermoscopy AK grading. Grade 1 AK presents focal areas of atypical honeycombed pattern at the level of the stratum spinosum, intermingled with areas of preserved, typical honeycombed pattern. In grade 2 AK, the atypia of the keratinocytes is more diffuse, involving the stratum spinosum and granulosum. Keratinocytes present a marked atypia, with different cell sizes and shapes. Grade 3 AK is characterized by a markedly atypical honeycombed pattern with areas of partial disruption of the normal epidermal layers, defined as a disarranged pattern. Pleomorphic keratinocytes show a wide variability in cellular size and shapes, and irregular intercellular keratinocyte connections are detected (Figure 2).

Sun-damaged skin presents as an area of mottled pigmentation, so it can be a challenge even for expert dermatologists to discriminate benign pigmented macules (such as lentigo solari) to pigmented tumor, such as lentigo maligna, pigmented AK [20,34]. AKs are very common lesions of sun-damaged skin and their differential diagnosis, when pigmented, and an early melanoma can be complex [34].

Dermoscopy and RCM are noninvasive diagnostic methods that have been shown to improve the diagnostic accuracy of melanoma and non-melanoma skin cancer when used together in the clinical workflow [35,36,37]. However, in a recent study analyzing facial pigmented lesions, pAK revealed a striking similarity with LM on dermoscopy. Accordingly, the authors concluded that almost all dermoscopic findings of LM can be also seen in pAK [22].

RCM has been shown to have an impact in the diagnosis of pigmented facial macules [35,38]. Moscarella et al. [38] suggest that an atypical honeycombed pattern with pleomorphic and atypical keratinocytes is a key feature of pAK. This pattern has also been previously reported for non-pigmented AK [34]. Moreover, they noticed frequently an increased epidermal thickness and intraepidermal dendritic cells referable to LC. The main confounding feature in the study by Hashemi et al. was the frequent detection of intraepidermal dendritic cells at RCM [39]. One of the most relevant RCM criteria for melanoma diagnosis is the presence of pagetoid, intraepidermal cells, either showing a dendritic or rounded shape [39,40] where rounded shaped cells are more specific for melanoma. In pAK the intraepidermal cells were morphologically characterized by short dendrites with no complex branching suggestive of nesting. When visible, the body of the cell was variably stellate. When considering RCM findings at the level of the DEJ, no features suggestive of melanocytic lesions were observed, and, as a peculiar finding, the detection of well-defined bright papillae associated with enlarged interpapillary space.

Due to an often clinically similar appearance, distinction between SCC and AK is still a challenge. Currently, the diagnostic distinction between AK and SCC skin lesions, especially when solely based on clinical aspects, may not always be reliable [41]. Collecting biopsies for histopathological analyses is however invasive and the feasibility is sometimes limited, mainly because of the risk of sampling errors. It was therefore necessary to search for in vivo features that are specific enough to discern between AK and SCC [42], and thereby better selecting the uncertain lesions for biopsy.

Unfortunately, the penetration depth imposes a major limitation on RCM in hyperkeratotic lesions, which are often associated with invasive SCC. The failure of RCM to provide images at the level of the dermoepidermal junction and stratum basale in potentially malignant hyperkeratotic lesions excludes important information from the diagnostic process. Instead, a distinction between SCC and AK using RCM must rely on the fact that, in SCC, extensive keratinocytic atypia involves the entire epidermis, including the stratum granulosum [30,42,43,44].

In AK, on the other hand, the stratum granulosum shows focally disarranged, mildly atypical or normal keratinocytes [42,43]. However, differentiation between SCC and AK remains challenging in hyperkeratotic lesions without visualization beyond the dermoepidermal junction [32]. In SCC, nest-like structures and pleomorphic cells can be detected in the dermis, while these RCM features are absent in AK [30,42]. Hyperkeratotic lesions differential diagnosis can therefore be difficult to obtain with noninvasive diagnostic techniques, and often require histopathological diagnostic confirmation.

## 6. Optical Coherence Tomography Aspects of AKs

OCT is an in vivo, noninvasive imaging technique based on interferiometry. Imaging is based on the use of infrared light, which permits an axial and lateral resolution of approximately 15 lm and a penetration depth of 500–1000 mm. The images are 2-dimensional, cross-sectional and have a lateral dimension of 4 to 6 mm [16]. Layers of skin, adnexal structures, and blood vessel can be detected, but the basement membrane and cellular and subcellular detail cannot [16]. OCT of the epidermis evidenced an 86% sensitivity and 83% specificity for the detection of AK compared with dermatologic assessment, which had sensitivity and specificity of 98% and 62%, respectively [16]. Preliminary studies have described the features of non-melanoma skin cancer including basal cell carcinoma (BCC) and AK and suggest that this technique may aid in the evaluation of NMSC [45,46].

A pilot study involving 20 participants was created to compare the appearance of normal skin and sun-damaged skin/early AK in OCT. Images of normal skin were recorded from the upper inner arm and sun-damaged skin/early AK from the dorsal forearm. OCT images of normal skin showed skin layers and features (stratum corneum, epidermis, dermis, blood vessels) similar to those previous reported in literature, while sun-damaged skin was characterized by increased signal in the epidermis and rapid attenuation of light. AKs were characterized by high surface reflection, the presence of a low-signal band in the stratum corneum, and heterogeneous appearance in the epidermis/dermis [45].

Conventional OCT allows real-time, in vivo examination of non-melanoma skin cancer. A new high-definition (HD)-OCT device is now available with an improved resolution and the ability to provide vertical (slice-mode) and horizontal (en-face mode) imaging. The en face mode is similar to the horizontal RCM scanning mode, and has been used to evaluate citological features of the examined lesion [47].

In earlier studies using conventional OCT, AKs were defined by the destruction of the epidermal/dermal layering and the presence of white streaks, dots and grey areas corresponding to hyperkeratosis [48].

Furthermore, thinning of epidermis and disruption of the entrance signal were described in the OCT imaging of AK. Barton et al. [46] found a dark band in the stratum corneum (SC) characteristic for AK with a sensitivity of 79% and a specificity of 100% with conventional OCT. However, in an observer-blinded study by Mogensen et al. [49] the sensitivity of recognizing AK and BCC in regular OCT was only 46%. They found that the OCT diagnosis was less reliable than the clinical diagnosis concerning BCC and AK, but the pathological lesions could be distinguished from healthy skin.

Maier et al. [50] conducted an AK study using HD-OCT and they found that there were distinct features present in the en-face mode of HD-OCT imaging of AK. The features identified were cellular and nuclear polymorphism, which to date, have not been described in conventional OCT [51].

Aghassi et al. [42] first described common RCM features for AK: presence of enlarged, pleomorphic nuclei varying in size, shape and orientation contrasting with small, uniform nuclei in AK compared to normal skin. Ulrich et al. later redefined diagnostic parameters to include superficial disruption, architectural disarray, and cellular polymorphism at the granular and spinous layer [16]. In RCM, normal skin shows a regular honeycombed pattern, described as roundish structures with dark nuclei and bright cytoplasm forming a regular web [50]. This description fits with the en-face mode HD-OCT image of stratum granulosum/stratum spinosum (SG/SS), although the differences are not as prominent as in RCM. Even though HD-OCT is not as precise as RCM, it is possible to detect several large dark roundish bodies with a bright irregular center, corresponding in histology to dyskeratotic cells. At RCM, dyskeratotic keratinocytes, typical of SCC, are described as large round bright cells with a dark nucleus [52], whereas Terhorst et al. [53] found similar cells, described as large cells with a bright center and a dark halo, possible corresponding to apoptotic cells in wound healing. Maier et al. [50] presented dyskeratotic and/or apoptotic with different discretions upon HD-OCT and RCM. Dyskeratotic and/or apoptotic cells show a condensed nucleus in HD-OCT images, while in RCM images are presented as dark cells with a bright center.

Early diagnosis and an accurate classification of AK is important for correct treatment. Nowadays clinicians can choose between lesion-directed or field-directed treatment [53]. Therefore, the importance of these new noninvasive diagnostic techniques increases day by day [54]. However, RCM and OCT are still not able to unequivocally detect an SCC from an AK, or distinguish subclinical lesions in the context of field cancerization.

The absence of an outlined DEJ was considered a highly sensitive morphological feature for SCC [47]. However, in 2015, Boone et al. [55] showed that 14% of AK also present a non-outlined DEJ, lowering considerably the specificity of the absence of an outlined DEJ for SCC diagnosis.

**Figure 3:**
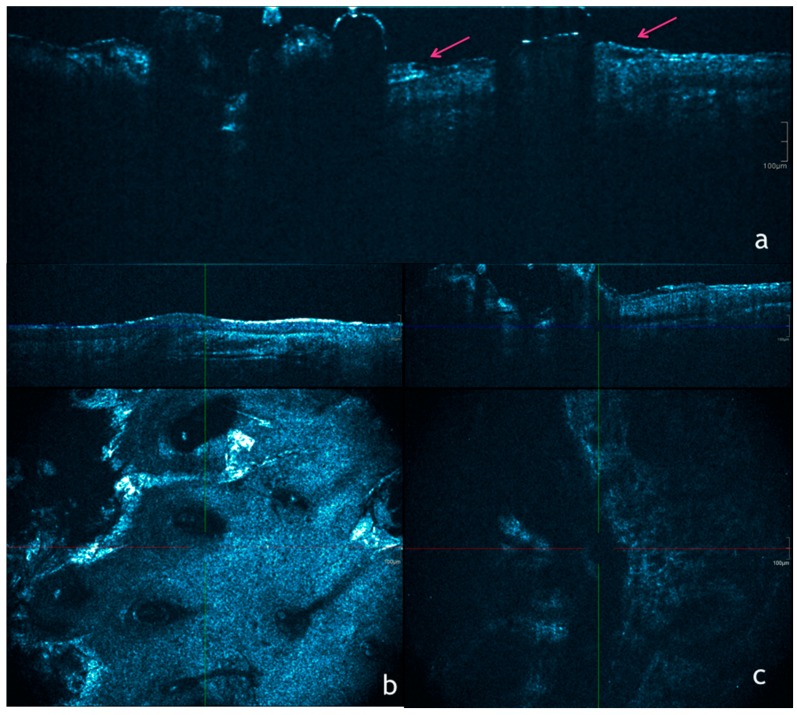
Optical coherence tomography of AKs. (**a**) Conventional OCT image showing point of disruption of the stratum corneum (pink arrows) which are typical of AKs; (**b**) En-face mode of HD-OCT showing mild atipya with superficial disruption, architectural disarray, and cellular polymorphism at the granular and spinous layer; (**c**) En-face mode of HD-OCT showing a very severe atypical honeycomb pattern and disarranged epidermal pattern due to the presence of dyskeratotic keratinocytes. These cells, are represented by round, bright nucleated cells.

Consequently, a relatively high number of unnecessary surgical interventions (moderate positive predictive value score of 76%) would be performed based only on the presence/absence of this criterion, thus making the question arise whether diagnostic accuracy could be improved by tissue optical properties analysis. Tissue optical analysis by HD-OCT seemed to enable assessment of discrete alterations in photodamaged skin (subclinical lesions) as well as more objective grading of clinical AK lesions [56]. To stratify the degrees of epidermal dysplasia, a three-tiered grading scale has been proposed by Cockerell et al. for AKs, which compares to the evaluation used for cervical dysplasia [57]. The localized epidermal atypia in AKs reflects a partial disruption of the differentiation program, whereas a more complete disruption of differentiation is associated with SCC in situ. Based on RCM diagnostic AK features, a good correlation with the histopathological AK grading was found with HD-OCT en face features [47,58]. These features were atypical honeycomb pattern (AHP) and/or disarranged epidermal pattern (DEP) due to cellular pleomorphism. In early or subclinical lesions, a mild AHP was confined to the bottom third of the epidermis. This finding correlated with KIN-I grading. Clinical AKs with an AHP involving the lower two-thirds of the epidermis were graded KIN-II. A full thickness DEP was only observed in AKs graded KIN-III. This DEP corresponded most probably with the presence of dyskeratotic keratinocytes (Figure 3). SCC presents a higher OCT signal, probably due to the presence of dyskeratotic cells. Assessment of follicular involvement is crucial because of the increased risk of progression to invasive SCC [56].

## 7. Conclusions

A number of noninvasive diagnostic methods are nowadays available for assistance in AK diagnosis. The obvious advantage of noninvasive diagnostic tools is the option to examine tissue in vivo, potentially reducing the need for biopsy. Dermoscopy is extremely helpful in the diagnosis of melanoma and BCC but it is not as useful in AK diagnosis, especially in the case of pigmented AKs. While only used in specialized skin cancer centers, OCT together with in vivo RCM evaluations appear to have the greatest potential for clinical applicability and evaluations of AKs. The low resolution and high penetration of OCT may be reciprocated by the near cellular resolution of RCM, which is limited by a shallow penetration. Comparable to routine histology, RCM permits the morphologic differentiation by detecting cellular and architectural patterns on horizontal sections. Furthermore, therapeutic effects and the continuous events of healing after treatment may be documented. In this regard, RCM may allow the therapeutic monitoring of patients with AK and assessment of efficacy, potentially detecting subclinical or residual disease, where previously only clinical evaluation was employed.

Histology remains the gold standard for skin cancer diagnosis, and consequently also for AK diagnosis. However, a biopsy represents a sample of a single lesion, usually about 4 mm. As a consequence, especially for larger lesions, the biopsy sampling may not be representative of the entire lesion. In the presence of two histologically different pathologies within the same lesion, the fact that only a small sample of tissue is excised, an incorrect diagnosis and subsequent unsuitable therapy can be the result. In most cases the operator decides to excise the biopsy in the area considered most representative within the lesion; this decision is usually guided by dermoscopy but does not exclude evaluation error. RCM and OCT enable in vivo and noninvasive evaluation of the whole lesion. The study of the entire lesion makes the diagnosis more certain and consequently allows the best treatment choice.
